# Gut microbiota of preterm infants supplemented with probiotics: sub-study of the *ProPrems* trial

**DOI:** 10.1186/s12866-018-1326-1

**Published:** 2018-11-13

**Authors:** Erica L. Plummer, Dieter M. Bulach, Gerald L. Murray, Susan E. Jacobs, Sepehr N. Tabrizi, Suzanne M. Garland, for the ProPrems Study Group, S. M. Garland, S. E. Jacobs, J. M. Tobin, S. N. Tabrizi, M. Pirotta, S. Donath, G. F. Opie, M. L. K. Tang, C. J. Morley, L. Hickey, K. Tan, A. Lewis, A. Veldman, J. Travadi, I. M. R. Wright, D. A. Osborn, J. Sinn, J. Levison, J. A. Stack, A. G. DePaoli, N. C. Austin, B. A. Darlow, J. M. Alsweiler, M. J. Buksh

**Affiliations:** 1 The Royal Women’s Hospital, Parkville, VIC 3052 Australia; 2 Murdoch Children’s Research Institute, The Royal Children’s Hospital, Parkville, VIC 3052 Australia; 3 Infection and Immunity Program, Monash Biomedicine Discovery Institute and Department of Microbiology, Monash University, Clayton, VIC 3800 Australia; 4 The University of Melbourne, Parkville, VIC 3050 Australia; 5 Victorian Life Sciences Computation Initiative, The University of Melbourne, Parkville Campus, LAB-14, 700 Swanston St, Carlton, VIC 3053 Australia; 6 The Royal Children’s Hospital, 50 Flemington Rd, Parkville, VIC 3052 Australia

**Keywords:** Probiotics, Very preterm infants, Gut microbiota, Necrotizing enterocolitis

## Abstract

**Background:**

The *ProPrems* trial, a multi-center, double-blind, placebo-controlled randomized trial, previously reported a 54% reduction in necrotizing enterocolitis (NEC) of Bell stage 2 or more from 4.4 to 2.0% in 1099 infants born before 32 completed weeks’ gestation and weighing < 1500 g, receiving probiotic supplementation (with *Bifidobacterium longum* subsp. *infantis* BB-02, *Streptococcus thermophilus* TH-4 and *Bifidobacterium animalis* subsp. *lactis* BB-12). This sub-study investigated the effect of probiotic supplementation on the gut microbiota in a cohort of very preterm infants in *ProPrems*.

**Results:**

*Bifidobacterium* was found in higher abundance in infants who received the probiotics (AOR 17.22; 95% CI, 3.49–84.99, *p* < 0.001) as compared to the placebo group, and *Enterococcus* was reduced in infants receiving the probiotic during the supplementation period (AOR 0.27; 95% CI, 0.09–0.82, *p* = 0.02).

**Conclusion:**

Probiotic supplementation with BB-02, TH-4 and BB-12 from soon after birth increased the abundance of *Bifidobacterium* in the gut microbiota of very preterm infants. Increased abundance of *Bifidobacterium* soon after birth may be associated with reducing the risk of NEC in very preterm infants.

**Electronic supplementary material:**

The online version of this article (10.1186/s12866-018-1326-1) contains supplementary material, which is available to authorized users.

## Background

Very preterm infants (born < 32 weeks’ gestation and weighing < 1500 g) are at increased risk of late-onset sepsis and necrotizing enterocolitis (NEC), with the associated potential for lifelong adverse health effects or death [1]. Preterm infants have a different gut microbiota than term newborns, including reduced species diversity, higher numbers of *Enterobacteriaceae* (including *Klebsiella pneumoniae* and *Escherichia coli*) and *Clostridium difficile*, and reduced numbers of *Bifidobacteria* and *Lactobacilli* [2, 3]. These differences may be a result of host factors associated with immaturity as well as various environmental factors including delivery mode, reduced exposure to maternal microbiota, exposure to antibiotic treatment, reduced breastfeeding, and prolonged stays in neonatal intensive care units [2, 4].

Necrotizing enterocolitis is a devastating inflammatory disease of the intestine that affects approximately 7% of infants born weighing < 1500 g [5]. Up to 30% of affected infants die, with increased rates of neurodevelopmental impairment in surviving NEC-affected infants compared to their unaffected peers [6]. The etiology of NEC is unknown and the current consensus is that it is likely to be multifactorial [5, 7, 8]. Non-microbial factors such as intestinal immaturity, genetic predisposition, and hypoxia-ischemia may contribute to the development of NEC [5, 7]. As NEC outbreaks have been reported, some hypothesize that the condition is caused by an infectious agent, although to date none has been found [9, 10]. It is generally accepted that a disturbance of the normal gut microbiota is a contributing factor to the development of NEC [8, 11, 12], with various associated dysbioses including reduced microbial diversity [13, 14], delayed colonization of commensals including *Bifidobacterium*, *Bacteroidetes*, and Firmicutes (including Negativicutes and Clostridia), and increased abundance of Gammaproteobacteria (with reports of increased Enterobacteriaceae, *Klebsiella* and *Escherichia/Shigella* in NEC infants compared to healthy controls) [13, 15–17].

There is strong evidence supporting the use of probiotic prophylaxis for the prevention of NEC. A recently published meta-analysis of 20 randomized controlled trials investigating the use of probiotics for prevention of NEC in preterm infants reported a 49.1% reduction in risk of NEC in probiotic supplemented infants (risk ratio [RR] = 0.509; 95% CI, 0.385–0.672; *p* < 0.001) [18]. In the *ProPrems* trial, a placebo-controlled randomized trial of 1099 infants, our group reported a 54% reduction in NEC of Bell stage 2 or more from 4.4 to 2.0% in very preterm infants receiving a three-strain probiotic (relative RR = 0.46; 95% CI 0.23–0.93; *p* = 0.03 [19]. In contrast, a large multicenter study of 1315 preterm infants found no reduction in the incidence of NEC following supplementation with a single-strain probiotic (adjusted RR = 0.93; 95% CI 0.68–1.27 [20]). This could suggest that not all probiotics have equivalent efficacy in preventing NEC and further work is needed to better understand how and why some probiotic combinations work and others do not.

In this sub-study, we investigated the effect of probiotic supplementation on the development of the gut microbiota of preterm infants by examining the gut bacterial communities in a cohort of very preterm infants enrolled in the *ProPrems* trial [19].

## Methods

### Participants and specimen collection

*ProPrems* [19, 21] was a large multi-center, double blind, placebo controlled randomized trial where very preterm infants (born < 32 weeks’ gestation and weighing < 1500 g) were randomized to receive either a probiotic combination (*Bifidobacterium longum* subsp. *infantis* (*BB–02*, 300 × 10^6^), *Streptococcus thermophilus* (*TH–4,* 350 × 10^6^) and *Bifidobacterium animalis* subsp. *lactis* (*BB-12,* 350 × 10^6^) (ABC Dophilus Probiotic Powder for Infants; Solgar, Leonia, New Jersey) with 1 × 10^9^ total organisms per 1.5 g, in a maltodextrin base powder) or placebo (maltodextrin powder) once enteral feeds were commenced, until discharge from hospital or term corrected age. Stool swabs (or perianal swabs if the infant had not passed feces) were collected from Victorian *ProPrems* participants as close to the following time points as possible: prior to commencement of the study powder, after one, four and eight weeks of treatment, at six and 12 months of age corrected for prematurity. Due to logistic limitations, only infants enrolled at The Royal Women’s Hospital, Melbourne, Australia with at least one swab available were eligible for inclusion in this sub-study. A total of 253 swabs (nine perianal swabs and 244 fecal swabs) from 68 infants were available for this sub-study.

### DNA extraction, PCR amplification, and pyrosequencing

DNA was extracted from the specimens using the MagNA Pure 96 System (Roche Diagnostics, Branchburg, NJ); stool swabs and perianal swabs were processed in the same way. The extracted DNA was used to generate an amplicon based library using *bifidobacteria* optimized PCR primers that amplify the V3-V5 hypervariable regions of the 16S rRNA gene as described by Sim et al. [22]. Sequencing of amplicons was performed on a Roche 454 Genome Sequencer instrument (GS FLX Titanium Chemistry) at Macrogen Inc. (Seoul, South Korea) generating single end reads. Multiplex Identification tags were incorporated during preparation.

### Sequence analysis

Sequence analysis was performed with QIIME (Version 1.8.0). Reads shorter than 250 bases, with homopolymer base runs of more than eight bases, containing more than eight ambiguously called bases or with an average phred quality score of less than 25 were removed from read sets. Chimeric reads were removed using UCHIME [23] in conjunction with the ‘gold’ 16S rRNA database [24]. Operational taxonomic unit (OTU) picking was performed using the default UCLUST algorithm and a similarity threshold of 97%. A representative sequence for each OTU was used to assign taxonomy, using the default UCLUST consensus taxonomy assigner and SILVA reference database [25]. Specimens with fewer than 100 reads following quality control were excluded from analysis.

### Data analysis

All statistical analyses and diversity calculations were completed with R Studio (version 0.98.1103, Boston, USA) employing R 3.2.0 [26].

Baseline characteristics were compared between allocation groups using the Chi-Square test or Fisher’s exact test for categorical variables, and the Wilcoxon rank sum test for continuous variables.

To test if bacterial abundance differed significantly between the allocation groups and with age, a logistic regression analysis using a mixed effects model was performed for each genus that had a mean abundance of at least 1% in one allocation group. Proportional abundances of each genera were converted to a binary variable (based on the median value) and were regressed against allocation, adjusting for age at sampling (i.e. time from birth; expressed in days as a continuous variable) and gestation (expressed as above or below 28 weeks’ gestation as a binary variable), clustering by participant number to account for multiple specimens from the same infant.

The prevalence of key bacteria was calculated in two ways: 1) the number of infants who had at least one specimen over the study period test positive for a specific genus as a proportion of the total number of infants in each allocation group, 2) the number of specimens positive for a specific genus as a proportion of the total number of specimens in each allocation group.

Bacterial diversity was defined as effective number of genera (which is the exponential of the Shannon diversity index) and was calculated using the vegan package [27]. Reads that could not be classified to a genus level were omitted from diversity analyses. Effect of probiotic treatment on diversity was examined using a mixed effects linear regression model, adjusting as above. All regression analyses were implemented using the lme4 package [28]. *P*-value false discovery rate adjustment for multiple testing was performed where required using the Benjamini-Hochberg method. An adjusted *p*-value < 0.05 was deemed significant.

A heatmap and associated dendrogram was generated using the vegan and gplots packages [27, 29] and were based on Bray-Curtis dissimilarities and Ward’s method for hierarchical clustering.

## Results

### Specimen and participant data

From the 253 specimens eligible for processing from 68 infants, a total of 1,064,333 reads were generated. Thirty-eight read sets (from a total of 30 infants) were excluded from analysis; twelve specimens failed to produce a PCR product and were not sequenced, and 26 specimens had fewer than 100 reads following quality control. The remaining 215 read sets (seven perianal swabs and 208 fecal swabs) from 66 infants (730,861 reads) were analyzed using QIIME. The median number of reads per specimen was 3263 (IQR = 1665–4657) and did not differ between the probiotic (median 3296 reads) and placebo group (median 3076 reads; Z = − 0.87, *p* > 0.05; Table 1). The probiotic group comprised 124 specimens from 38 infants and the placebo group comprised 91 specimens from 28 infants. A range of one to five specimens was obtained from each infant (average three specimens (SD = 0.8)). Details of specimen collection are in Table 1.

**Table 1:** Overview of study participants

	Probiotic, *n* = 38	Placebo, *n* = 28	*P* value^a^
Demographics			
Male, n (%)	19 (50.0)	14 (50.0)	1.0
Gestational age, wk., mean (SD)	28.6 (1.81)	27.5 (1.72)	0.02
Birth weight, g, mean (SD)	1040 (285)	1000 (253)	0.53
Maternal antibiotics, n (%)^b^	20 (52.6)	12 (42.9)	0.43
Infant antibiotics:			
Courses of antibiotics, median (IQR))	1 (0–2)	1 (0–2)	0.48
Days of antibiotic exposure, d, median (IQR)	2 (0–8)	5 (0–10)	0.38
Infants with at least 1 episode of definite late-onset sepsis with pathogen, n(%)^c^	3 (7.9)	2 (7.1)	1.0
Infants with at least 1 episode of definite late-onset sepsis with CoNS, n(%)^c^	2 (5.3)	4 (14.3)	0.39
Caesarean delivery, n (%)	27 (71.1)	19 (60.7)	0.78
Any breast milk feeding, n (%)	37 (97.4)	28 (100.0)	1.0
Age commenced study powder, d, median (IQR)	3.0 (2.0–5.0)	3.5 (2.0–5.5)	0.51
Age finished study powder, d, median (IQR)	67.5 (54.0–85.0)	73.0 (64.0–89.5)	0.14
Length of supplementation, d, median (IQR)	62.5 (48.5–81.8)	69.5 (62.0–83.3)	0.17
Sequencing data			
Specimens included, n	124	91	
Specimens collected before supplementation commenced, n	3	8	
DOL, median (range)	3 (3–4)	4 (1–8)	
Specimens collected during supplementation period, n	80	62	
DOL, median (range)	30 (6–72)	31 (4–96)	
Specimens collected post supplementation period, n	41	21	
DOL, median (range)	256 (58–529)	285 (61–613)	
Specimens collected per baby, mean (SD)	3 (0.8)	3 (0.8)	
Number of reads per specimen, median (IQR)	3296 (1729–5136)	3076 (1654–4472)	0.39

*Abbreviations: DOL* Day of life of specimen collection, *IQR* interquartile range, *CoNS* Coagulase-negative *Staphylococcus*, *SD* standard deviation

^a^P value is based on Chi-Square test or Fisher’s exact test for categorical variables and Wilcoxon rank sum test for continuous variables. ^b^ maternal antibiotics is presented as the number (%) of mothers reporting antibiotic use before or during labor. ^c^ late-onset sepsis > 48 h after birth and before discharge home or term postmenstrual age

Baseline patient demographic characteristics were similar between the probiotic and placebo group, except that gestational age was lower in the placebo group (*p* = 0.02) (Table 1). The 66 infants included in this sub-study are representative of the entire *ProPrems* cohort with regard to the demographic characteristics including gender, gestational age, birth weight, and caesarean delivery (Additional file 1). However, the rate of NEC was lower in the sub-study population (one infant included in this sub-study developed NEC (Bell Stage 2 or more) (1.5%), compared to 35 infants (3.2%) in the wider *ProPrems* cohort).

### The impact of probiotic supplementation on the gastrointestinal microbiota

A total of 102,050 reads (14.0%) could not be assigned to a genus; this included 400 reads that could not be assigned any bacterial phylum. The majority of reads that could not be classified to a bacterial genus were from the *Enterobacteriaceae* family (60.5%).

*Bifidobacterium* was the most prevalent genus in specimens from probiotic supplemented infants (detected in 90% of specimens collected from probiotic infants vs 55% of specimens collected from control infants). *Streptococcus* was present in 75% of specimens collected from probiotic infants vs 53% of specimens collected from control infants. *Enterobacter* was the most prevalent genus in specimens from control infants (detected in 85% of specimens collected from control infants vs 77% of specimens collected from probiotic infants). *Bifidobacterium* and *Streptococcus* were detected in at least one specimen from all probiotic supplemented infants; conversely, *Enterobacter*, *Escherichia/Shigella* and *Enterococcus* were detected in at least one specimen from all control infants **(**Table 2**)**.

**Table 2:** Logistic mixed model regression analysis for examining the effect of probiotic supplementation on bacterial genera abundance

	Probiotic (n = 38 infants, 124 specimens)		Placebo (n = 28 infants, 91 specimens)				
Genus^a^	Prevalence n infants (%)^b^; n specimens (%)^c^	Relative abundance Mean % (SD)	Prevalence n infants (%)^b^; n specimens (%)^c^	Relative abundance Mean % (SD)	AOR^d^ (95% CI)	*P* value	Adjusted *P* value^e^
*Bifidobacterium*	38 (100); 111 (90)	36.4 (32.5)	25 (89); 50 (55)	17.5 (27.4)	4.28 (2.02–9.10)	**< 0.001**	**0.002**
*Enterobacter*	37 (97); 95 (77)	14.8 (24.6)	28 (100); 77 (85)	18.7 (25.1)	0.75 (0.40–1.41)	0.37	0.45
*Escherichia/Shigella*	37 (97); 84 (68)	9.1 (19.1)	28 (100); 74 (81)	7.5 (17.2)	0.69 (0.34–1.43)	0.32	0.43
*Staphylococcus*	36 (95); 65 (52)	8.1 (24.0)	24 (86); 48 (53)	7.5 (20.4)	0.85 (0.47–1.55)	0.60	0.60
*Enterococcus*	37 (97); 76 (61)	3.7 (11.0)	28 (100); 71 (78)	8.8 (19.3)	0.37 (0.20–0.71)	**0.003**	**0.02**
*Streptococcus*	38 (100); 93 (75)	4.7 (15.0)	25 (89); 48 (53)	2.9 (9.4)	1.57 (0.88–2.80)	0.13	0.31
*Veillonella*	32 (84); 56 (45)	2.4 (7.2)	25 (89); 46 (51)	3.3 (7.9)	0.74 (0.42–1.33)	0.32	0.43
*Clostridium*	32 (84); 53 (43)	0.5 (2.4)	27 (96); 45 (49)	3.0 (9.0)	0.68 (0.38–1.21)	0.19	0.31
*Lactobacillus*	23 (61); 41 (33)	0.9 (3.7)	13 (46); 19 (21)	2 (7.6)	1.85 (0.78–4.41)	0.16	0.31
*Citrobacter*	20 (53); 27 (22)	0.8 (3.4)	21 (75); 34 (37)	1.7 (10.1)	0.44 (0.19–1.02)	0.06	0.21
*Akkermansia*	6 (16); 6 (5)	0.1 (0.5)	3 (11); 3 (3)	1.4 (9.2)	1.87 (0.41–8.59)	0.42	0.47
*Pantoea*	11 (29); 14 (11)	0.1 (0.8)	12 (43); 18 (20)	1.1 (10.1)	0.45 (0.15–1.35)	0.15	0.31

*Abbreviations: AOR* adjusted odds ratio, *CI* confidence interval, *IQR* interquartile range; *P* values <0.05 are bolded to indicate statistically significant associations.

^a^Proportional abundances of each genera were converted to a binary variable (based on the median value). Only genera that had a mean abundance of at least 1% abundant in one (or both) allocation group were included in regression analysis; ^b^ Presents the number (and percent) of infants who had at least on specimen over the study period test positive for genus; ^c^ Presents the number (and percent) of total specimens test positive for genus ^d^ Odds ratio for mixed effects regression model association between allocation group and bacterial abundance adjusted for gestation and age at sampling, clustering by participant number to account for multiple specimens from infants (66 clusters). ^e^ *P*-value false discovery rate adjustment for multiple testing was performed using the Benjamini-Hochberg method

**Fig. 1:**
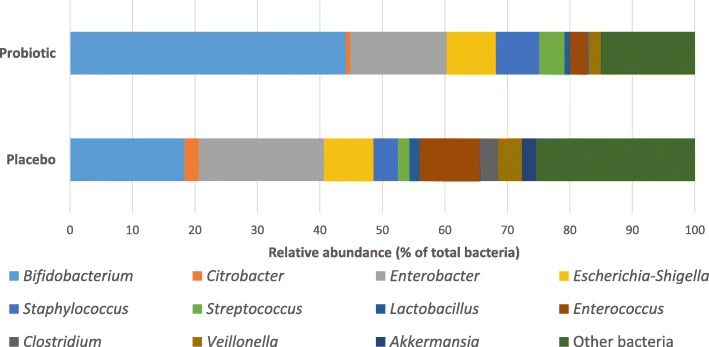
Compares the percent abundance of specific genera between the probiotic and placebo infants. The 12 genera included had a mean abundance of at least 1% in one (or both) allocation group and were included in the regression analysis. Bacteria not included in the regression analysis are grouped under “Other bacteria”

Twelve genera had a mean abundance of at least 1% in one (or both) allocation groups (Fig. 1) and these were the focus for regression analyses. After adjusting for age and gestation, babies receiving the probiotic had an increased abundance of *Bifidobacterium* (AOR 4.28; 95% CI, 2.02–9.10, adjusted *p*-value =0.002) and decreased abundance of *Enterococcus* (AOR 0.37; 95% CI, 0.20–0.71, adjusted p-value =0.02). Despite being detected more frequently in probiotic supplemented infants compared to control infants no significant difference in the abundance of *Streptococcus* was observed between allocation groups after adjusting for age and gestation (AOR 1.57; 95% CI, 0.88–2.79, adjusted *p*-value > 0.05; Table 2). Additional file 2 provides a graphical representation of the relative abundance of the 20 most abundant genera found in study specimens. Abundance data at the genus level for each specimen is provided in Additional file 3.

Given these findings, additional post-hoc logistic regression analyses were conducted. Specimens were grouped into two categories: collected during the supplementation period and collected post supplementation period. Supplementation on average started at four days of age and stopped at 68 days from birth. Eleven specimens collected before supplementation commenced were excluded from this analysis. *Bifidobacterium* and *Enterococcus* were regressed against allocation, adjusting for age, gestation and multiple specimens from each infant as above. The difference in abundance of *Bifidobacterium* and *Enterococcus* between the allocation groups was only evident during the supplementation period (AOR 17.22; 95% CI, 3.49–84.99, *p* < 0.001 and AOR 0.27; 95% CI, 0.09–0.82, *p* = 0.02 respectively) (Table 3).

**Table 3:** Logistic mixed model regression analysis for examining the effect of probiotic supplementation on abundance of *Bifidobacterium* and *Enterococcus* during and following the supplementation period

	Specimens collected during supplementation period *N*=142^a^				Specimens collected post supplementation period *N*=62^a^			
Genus	Probiotic % abundance mean (SD) (*N* = 80)	Placebo % abundance mean (SD) (*N* = 62)	AOR^b^ (95% CI)	*P* value	Probiotic % abundance mean (SD) (*N* = 41)	Placebo % abundance mean (SD) (*N* = 21)	AOR^c^ (95% CI)	*P* value
*Bifidobacterium*	40.5 (31.9)	14.4 (26.7)	17.22 (3.49–84.99)	< 0.001	28.7 (31.2)	26.9 (27.0)	0.99 (0.30–3.26)	0.99
*Enterococcus*	3.3 (8.9)	10.0 (20.8)	0.27 (0.09–0.82)	0.02	4.9 (14.6)	1.7 (1.9)	0.36 (0.11–1.17)	0.09

*Abbreviations: AOR* adjusted odds ratio, *CI* confidence interval, *IQR* interquartile range;

^a^A total of 204 specimens were included in this analysis. Specimens collected prior to the supplementation commenced (*n* = 11) were excluded from this analysis; ^b^ Odds ratio for adjusted mixed effects regression model for association between allocation group and bacterial abundance during the supplementation period, clustering by participant number to account for multiple specimens from infants (63 clusters); ^c^ Odds ratio for adjusted mixed effects regression model association between allocation group and bacterial abundance following the supplementation period, clustering by participant number to account for multiple specimens from infants (51 clusters)

Of note several genera were more abundant at different ages. *Staphylococcus* and *Enterobacter* were found in higher abundance in specimens collected closer to birth, compared to those collected later. *Veillonella*, *Clostridium*, *Streptococcus, Akkermansia* and *Citrobacter* were found in higher abundance in specimens collected later (Additional file 4).

No difference in effective number of genera was observed between the probiotic and placebo groups (adjusted coef − 0.3; 95% CI, − 0.7-0.1, *p* > 0.05).

## Discussion

This sub-study investigated the effect of probiotic supplementation with *B. longum* subsp. *infantis* BB-02, *S. thermophilus* TH-4 and *B. animalis* subsp. *lactis* BB-12 on the gut microbiota of a subset of very preterm infants enrolled in the *ProPrems* trial and found that infants supplemented with probiotics had a higher abundance of *Bifidobacterium* compared to control infants.

*ProPrems* demonstrated that probiotic supplementation with BB-02, TH-4 and BB-12 resulted in a 54% relative risk reduction in NEC of Bell stage 2 or more [19]. However, as the incidence of NEC in the *ProPrems* control infants was low (4.4%) and only one infant who developed NEC was included in this sub-study, few conclusions can be made about the mechanism/s by which the probiotics may work to prevent NEC. Nevertheless, it is noteworthy that the increased abundance of *Bifidobacterium* in probiotic-supplemented infants was only observed during the supplementation period. This may suggest that increased abundance of *Bifidobacterium* when the gut and immune system are most immature is important in reducing the risk of NEC in very preterm infants.

*Bifidobacterium* spp. are known inhabitants of the adult and full term infant gut, but their presence is often reduced or delayed in preterm infants [30, 31]. Probiotic supplementation with *Bifidobacterium* spp*.* has been shown to promote colonization of *Bifidobacterium* spp. in preterm and low birth weight infants [20, 32–36], often resulting in positive effects including weight gain, decreased intestinal permeability, reduction in abundance of potentially pathogenic bacteria, and establishment of a gut microbiota similar to that of healthy full term infants [33, 35, 36].

Interestingly, the *PiPs Study* found no reduction in the incidence NEC in infants supplemented with *Bifidobacterium breve* BBG-001, despite confirmation of *B. breve* colonization by culture and qPCR in 85% of probiotic supplemented infants and 37% of control infants at 2 weeks postnatal age [20]. The results of the *PiPs Study* highlight the importance of *Bifidobacterium* strain/s selection in developing an effective probiotic for preventing NEC [37]. Given the multifactorial nature of NEC, and that different bacterial strains are thought to exert probiotic effects by different mechanisms, multi-strain probiotics may be more beneficial than single-strain formulations in the prevention of NEC [38]. This is supported by a recent meta-analysis that reported a lower incidence of NEC in infants supplemented with multi-strain probiotics compared with infants supplemented with single strain combinations using *Lactobacillus* spp., *Bifidobacterium* spp. and *Saccharomyces boulardii* [39].

Despite detecting a difference in the abundance of *Bifidobacterium* between the allocation groups, no similar effect was observed for *Streptococcus*. This is despite the probiotic comprising both *Bifidobacterium* and *Streptococcus*, and the prevalence of *Streptococcus* being higher in the probiotic group compared to the placebo group. This may indicate that *Bifidobacterium* has a more intimate interaction with the gut mucosa than *Streptococcus* [40, 41]. The increased abundance of *Bifidobacterium* compared to *Streptococcus* could also be a result of the greater quantity of *Bifidobacterium* in the probiotic formulation compared to *Streptococcus*.

Excluding *Bifidobacterium*, the most abundant bacterial taxonomic groups identified were the genera *Enterococcus*, *Staphylococcus* and *Streptococcus* and the Enterobacteriaceae family, which is consistent with current literature for preterm infants [32, 42, 43]. *Enterococcus* was the only genus found in significantly higher abundance in the control infants. Enterococci are known colonizers of the preterm gut, and have been identified in both healthy infants and infants who go on to develop NEC [44–47].

This study has a number of limitations. First, 16S rRNA gene sequencing only allowed allocation of reads to genus level. As a result, it cannot be determined if the increase in *Bifidobacterium* in the probiotic-supplemented infants comprised the probiotic BB-12 and BB-02 strains. Furthermore, we observed an overall high abundance of *Bifidobacterium* in both allocation groups compared to other studies of the infant gut microbiota [13, 48, 49]. This could be a result of the *bifidobacteria* optimised primers used for 16S rRNA gene amplification, which have been shown to improve the amplification of *Bifidobacterium* in fecal samples [22]. Cross-colonization may also have accounted for the higher than expected abundance of *Bifidobacterium* observed in controls. We previously reported a low occurrence of probiotic cross-colonization of infants in a neonatal unit during and after the *ProPrems* study [50]. However, this study was limited by a small number of *ProPrems* participants (twelve of the 87 infants analyzed were *ProPrems* participants) and several studies have reported cross-colonization in up to 44% of control infants during and/or after supplementation [20, 33]. Future research utilizing a whole metagenome approach will provide species and strain level information, as well as identify whether cross-colonization or primer selection may have influenced the microbial profiles presented here.

Second, infants included in this study contributed variable numbers of specimens, so colonization patterns could not be established for all infants. Additionally, only eleven specimens collected before supplementation commenced were available for analysis. As a result, we do not know if there was a difference in the pre-supplementation gut microbiota between the two allocation groups. Finally, the 66 infants included in this sub-study represent only a small proportion of the 1099 total *ProPrems* study population. Though the infants in this sub-study are representative of the wider *ProPrems* cohort in terms of demographic details, they did have a lower incidence of NEC (Bell Stage 2 or more). Specimens from only one infant who developed NEC were available for inclusion in this sub-study. As such, we cannot compare the gut microbiota of infants who developed NEC with those who remained healthy.

## Conclusion

Probiotic supplementation with BB-02, BB-12, and TH-4 increased the abundance of *Bifidobacterium* in the gut microbiota of very preterm infants during probiotic supplementation. Increased abundance of *Bifidobacterium* shortly after birth may be protective against NEC. A detailed understanding of the impact of probiotic supplementation on the gut microbiota at a strain level is required given current research has highlighted that selecting the appropriate *Bifidobacterium* strain/s for probiotic supplementation in very preterm infants is crucial.

## Additional files

Additional file 1: Comparison of demographics of sub-study participants and the wider ProPrems cohort (DOCX 13 kb)

Additional file 2: Heatmap of bacterial abundance in all study specimens. Each vertical line represents the bacterial composition of one specimen. The 20 most abundant taxa found in specimens are included in the heatmap. Allocation group is displayed above the heatmap in red (probiotic) and blue (placebo). The stage of study is also displayed above the heatmap in purple (before supplementation), pink (during supplementation) and cyan (post supplementation). (PDF 116 kb)

Additional file 3: Relative abundance of all detected genera for each specimen (XLSX 120 kb)

Additional file 4: Logistic mixed model regression analysis for examining the effect of time from birth on bacterial genera abundance. (DOCX 16 kb)

## Abbreviations

AOR: Adjusted odds ratio
CI: Confidence interval
NEC: Necrotizing enterocolitis
rRNA: ribosomal RNA
