# Supplementary material for: Gut microbiota of preterm infants supplemented with probiotics: sub-study of the ProPrems trial
Source: BMC Microbiol. 2018 Nov 13;18:184. doi: 10.1186/s12866-018-1326-1 (PMC6234596; doi:10.1186/s12866-018-1326-1)
Supplement: Supplementary file 1 — Comparison of demographics of sub-study participants and the wider ProPrems cohort (DOCX 13 kb) [file 12866_2018_1326_MOESM1_ESM.docx]

**Additional File 1.** Comparison of demographics of sub-study participants and the wider ProPrems cohort

|  | **Biome sub-study**  **N=66** | **ProPrems**  **N=1099** |
| --- | --- | --- |
| **Demographics** |  |  |
| Male, n (%) | 33 (50.0) | 572 (50.0) |
| Gestational age, wk, mean (SD) | 28 (1.8) | 28 (2.0) |
| Birth weight, g, mean (SD) | 1023 (270) | 1056 (260) |
| Maternal antibiotics, n (%) | 32 (48.5) | 532 (48.4) |
| Infant antibiotics, n (%) | 40 (60.6) | 645 (58.7) |
| Caesarean delivery, n (%) | 46 (69.7) | 736 (67.0) |
| Any breast milk feeding, n (%) | 65 (98.5) | 1052 (95.7) |
| Age commenced study powder, d, median (IQR) | 3 (2-5) | 5 (4-7) |
| Age finished study powder, d, median (IQR) | 71.5 (55-89) | 67 (50-82) |
| Length of supplementation, d, median (IQR) | 65.5 (53-83) | 63 (46-78) |
| NEC Bell Stage 2 or more, n(%) | 1 (1.5) | 35 (3.2) |

Abbreviations: NEC, necrotising enterocolitis; IQR, interquartile range; SD, standard deviation
