# Supplementary figures and images for: Gut microbiota of preterm infants supplemented with probiotics: sub-study of the ProPrems trial
Source: BMC Microbiol. 2018 Nov 13;18:184. doi: 10.1186/s12866-018-1326-1 (PMC6234596; doi:10.1186/s12866-018-1326-1)

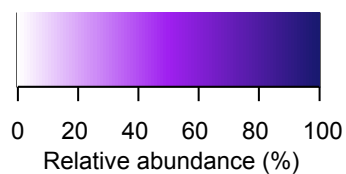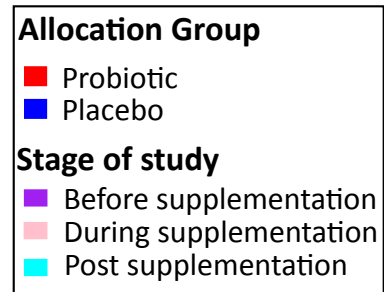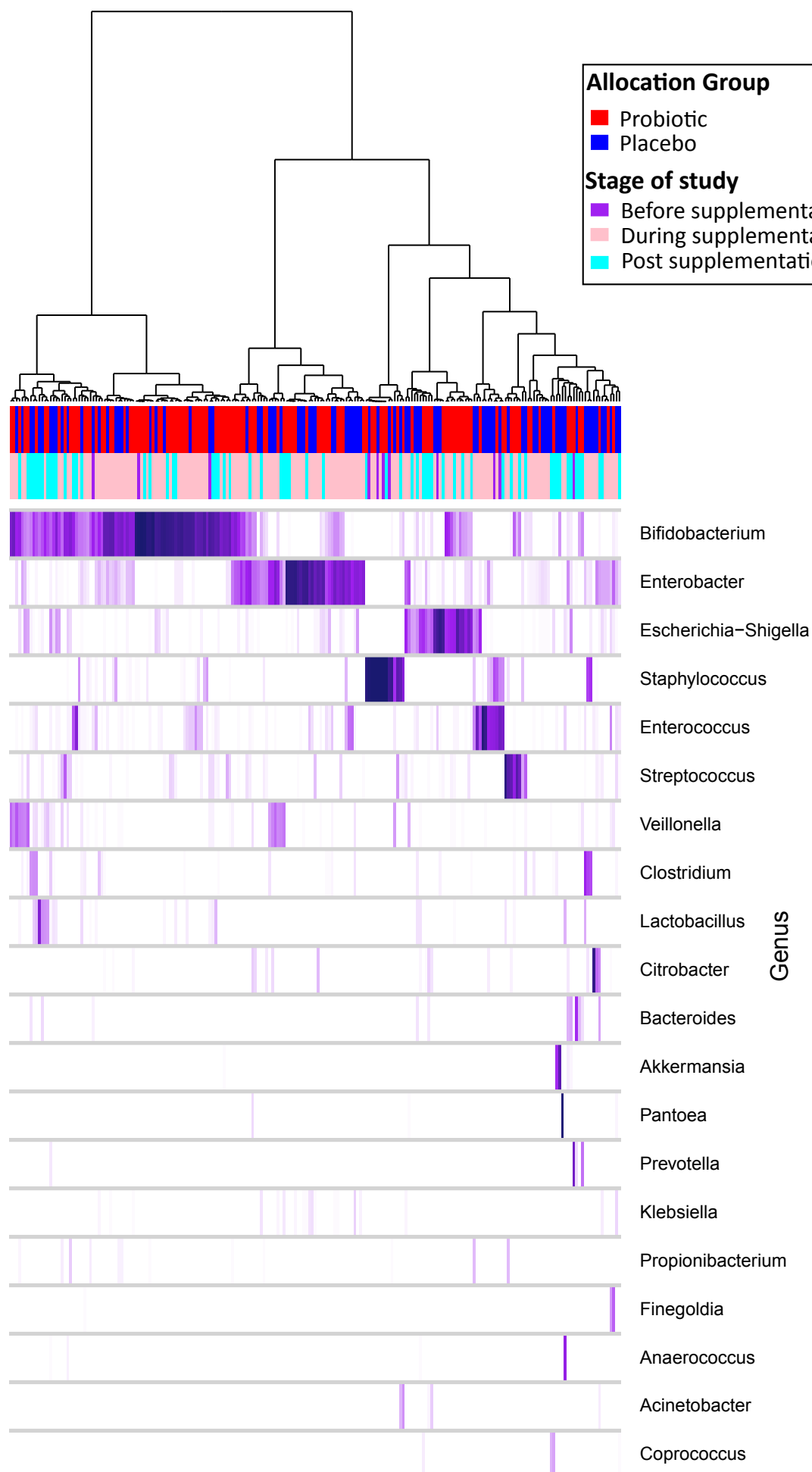

Supplement: Supplementary file 2 — Heatmap of bacterial abundance in all study specimens. Each vertical line represents the bacterial composition of one specimen. The 20 most abundant taxa found in specimens are included in the heatmap. Allocation group is displayed above the heatmap in red (probiotic) and blue (placebo). The stage of study is also displayed above the heatmap in purple (before supplementation), pink (during supplementation) and cyan (post supplementation). (PDF 116 kb) [file 12866_2018_1326_MOESM2_ESM.pdf]
