# Supplementary material for: Gut microbiota of preterm infants supplemented with probiotics: sub-study of the ProPrems trial
Source: BMC Microbiol. 2018 Nov 13;18:184. doi: 10.1186/s12866-018-1326-1 (PMC6234596; doi:10.1186/s12866-018-1326-1)
Supplement: Supplementary file 4 — Logistic mixed model regression analysis for examining the effect of time from birth on bacterial genera abundance. (DOCX 16 kb) [file 12866_2018_1326_MOESM4_ESM.docx]

**Additional File 4. Logistic mixed model regression analysis for examining the effect of time from birth on bacterial genera abundance**

| Genus ^a^  (N=66 participants, 215 specimens) | AOR ^b^  (95% CI) | Adjusted  P value ^c^ | Sensitivity analysis with perianal swabs excluded (N=66 participants, 208 specimens) | |
| --- | --- | --- | --- | --- |
|  |  |  | AOR ^d^  (95% CI) | Adjusted  P value ^c^ |
| *Bifidobacterium* | 1.001  (0.998-1.003) | 0.71 | 1.000  (0.998-1.003) | 0.84 |
| *Enterococcus* | 1.000  (0.998-1.002) | 0.99 | 1.000  (0.997-1.002) | 0.82 |
| *Citrobacter* | 1.004  (1.001-1.007) | **0.01** | 1.004  (1.001-1.006) | **0.02** |
| *Streptococcus* | 1.004  (1.001-1.006) | **0.01** | 1.004  (1.001-1.006) | **0.02** |
| *Lactobacillus* | 1.001  (0.999-1.004) | 0.41 | 1.001  (0.998-1.004) | 0.48 |
| *Clostridium* | 1.005  (1.002-1.007) | **0.002** | 1.004  (1.002-1.007) | **0.003** |
| *Pantoea* | 0.997  (0.993-1.002) | 0.30 | 0.997  (0.993-1.001) | 0.25 |
| *Escherichia/Shigella* | 1.003  (1.000-1.005) | 0.06 | 1.002  (1.000-1.005) | 0.16 |
| *Veillonella* | 1.005  (1.002-1.008) | **0.001** | 1.005 (1.002-1.008) | **0.002** |
| *Enterobacter* | 0.997  (0.994-0.999) | **0.03** | 0.996  (0.994-0.999) | **0.01** |
| *Akkermansia* | 1.007  (1.003-1.012) | **0.002** | 1.007  (1.003-1.011) | **0.004** |
| *Staphylococcus* | 0.992  (0.989-0.995) | **<0.001** | 0.992  (0.989-0.995) | **<0.001** |

Abbreviations: AOR, adjusted odds ratio; CI, confidence interval; IQR, interquartile range;

^a^ Proportional abundances of each genera were converted to a binary variable (based on the median value). Only genera that had a mean abundance of at least 1% abundant in one (or both) allocation group were included in regression analysis; ^b^ Odds ratio for mixed effects regression model association between time from birth and bacterial abundance adjusted for allocation and gestation, clustering by infant to account for multiple specimens from infants (66 clusters). ^c^ P-value false discovery rate adjustment for multiple testing was performed using the Benjamini-Hochberg method. ^d^ Odds ratio for mixed effects regression model association between time from birth and bacterial abundance adjusted for allocation and gestation with perianal specimens removed, clustering by infant to account for multiple specimens from infants (66 clusters).
